# Deep learning-based automated classification of endometrial lesions in IVF patients using hysteroscopic images

**DOI:** 10.1038/s41598-026-57032-0

**Published:** 2026-06-06

**Authors:** Marah Barakat, Rasha Massoud, Marwan Alhalabi

**Affiliations:** 1 Department of Biomedical Engineering, Faculty of Mechanical and Electrical Engineering, Damascus University, Damascus, Syrian Arab Republic; 2 Division of Reproductive Medicine, Embryology and Genetics, Faculty of Medicine, Damascus University, Damascus, Syrian Arab Republic

**Keywords:** Hysteroscopy, Endometrial polyps, Endometritis, Transfer learning, In vitro fertilization (IVF), Grad-CAM, Computational biology and bioinformatics, Diseases, Health care, Medical research

## Abstract

Endometrial health is a key determinant of female fertility and successful pregnancy outcomes, making the accurate diagnosis of endometrial lesions essential for the success of assisted reproductive technology. While hysteroscopy remains the gold standard for uterine cavity evaluation, interpretation can vary based on clinical expertise. To address this, we developed a deep learning-based clinical decision support system to classify hysteroscopic images from high-resolution (4 K) videos into three categories: normal endometrium, endometrial polyps, and endometritis. Endometritis cases were classified based on hysteroscopic features suggestive of inflammation; no histopathological confirmation was obtained. Utilizing a dataset of 1500 expert-annotated images from 200 clinical videos, we applied transfer learning across four architectures: VGG-16, VGG-19, DenseNet-121, and EfficientNet-B0. Our results show that the models achieved classification accuracies between 85 and 89%, with DenseNet-121 demonstrating superior performance, specifically achieving a sensitivity of 93% and an AUC of 98.8% for polyp detection, alongside a precision of 90% for endometritis. Furthermore, Grad-CAM visualization confirmed that the networks focused on clinically relevant morphological features, enhancing model interpretability. These findings suggest that deep learning may serve as a supportive tool to assist clinicians in hysteroscopic analysis, pending validation on external datasets and with pathologically confirmed labels.

## Introduction

Infertility is a common medical condition affecting a significant proportion of couples worldwide and has a substantial impact on their ability to conceive^1^.

For couples experiencing infertility, assisted reproductive technologies (ART) represent the optimal therapeutic approach, particularly in vitro fertilization (IVF), which is currently the most commonly used and effective procedure^2^.

The success of IVF depends not only on embryo quality but also on the health and receptivity of the endometrium which is considerably more complex than the morphological and molecular evaluation of embryo quality. Consequently, implantation failure—which affects 10–30% of patients—remains a major challenge despite advances in stimulation protocols and reproductive surgery^2,3^.

The endometrium functions as the “guardian of pregnancy,” permitting embryo implantation only within a narrow temporal window. Consequently, assessing endometrial receptivity is essential for optimizing embryo transfer timing and reducing the risk of implantation failure^3^. Abnormalities of the uterine cavity are observed in approximately 50% of women experiencing recurrent implantation failure, highlighting the critical importance of thorough uterine evaluation in infertility cases^1^.

Endometritis is an inflammatory condition of the endometrium that can present acutely with obvious clinical symptoms or chronically, often asymptomatic and detected during infertility evaluation. It has been associated with reduced pregnancy rates and increased risk of implantation failure^4^. Endometrial polyps (EPs), among the most common intrauterine lesions, adversely affect endometrial receptivity and contribute to infertility, frequently accompanied by abnormal uterine bleeding and pelvic pain, with a reported risk of malignant transformation ranging from 0–13%^2,5^. In clinical practice, hysteroscopic examination may reveal visual features suggestive of endometrial inflammation, including stromal oedema, focal or diffuse endometrial hyperaemia, the so-called “strawberry” appearance of the endometrium, and the presence of micropolyps^5^. Polyp presence is often linked to chronic endometritis, diagnosed histologically by the presence of plasma cells within the endometrial stroma, further negatively impacting IVF outcomes^2,6^. Hysteroscopic examination detects polyps in approximately 4% of women with unexplained infertility and 14.8% of women with regular menstrual cycles, underscoring their clinical relevance in pre-ART uterine assessment and preparation^7^.

Three-dimensional ultrasound imaging techniques are non-invasive and easy to use; however, they may fail to detect subtle intrauterine lesions such as small polyps, chronic inflammation, or mild adhesions^8^. Consequently, hysteroscopy is considered the gold standard for endometrial assessment, as it provides direct visualization and allows for tissue biopsy and therapeutic interventions. Nevertheless, it carries potential risks, including bleeding and uterine perforation, which are highly dependent on operator experience. Inexperienced hysteroscopists may unnecessarily prolong the procedure or overlook certain lesions, particularly small polyps, highlighting the need for supportive diagnostic tools to bridge this gap^9^.

Artificial Intelligence (AI) has revolutionized medical diagnostics by mimicking human cognitive functions to analyze complex clinical data^10^. Within this domain, Deep Learning (DL), a specialized subset of machine learning, employs multi-layered neural networks to autonomously extract hierarchical features from high-dimensional datasets. DL has demonstrated exceptional efficacy in medical imaging—spanning classification, enhancement, and segmentation—often identifying subtle pathological signatures that elude conventional human observation^11,12^.

While deep neural networks have achieved significant breakthroughs in gastrointestinal endoscopy and oncological screening^13,14^, their application in hysteroscopic imaging remains disproportionately limited. This scarcity of research is particularly evident in the context of patients undergoing Assisted Reproductive Technologies (ART). Despite the clinical gravity of endometrial assessment for IVF success, the integration of automated diagnostic tools in hysteroscopy is still in its infancy.

Existing literature has primarily focused on broad categorizations, such as distinguishing benign from malignant lesions using VGG-16^14,15^ or employing YOLOX for polyp detection^9^. However, these studies often overlook hysteroscopically-suspected endometritis—a frequently asymptomatic inflammatory condition that remains a critical factor in recurrent implantation failure. To our knowledge, no study has yet evaluated the synergistic use of DL and Explainable AI (XAI) to classify the triad of normal endometrium, polyps, and hysteroscopically-suspected endometritis specifically within an IVF population.

To address this gap, this study applies multiple state-of-the-art transfer learning models to a representative hysteroscopic image dataset of IVF patients to enhance the accuracy of endometrial lesion classification. While various convolutional neural network architectures have been explored in hysteroscopic image analysis, this research specifically introduces the application of DenseNet-121 to this clinical domain—an architecture that has remained largely unaddressed in previous studies. By leveraging its dense connectivity, we aim to explore whether such models can better capture the subtle pathological features of endometrial lesions compared to more commonly used frameworks. Additionally, explainable artificial intelligence (XAI) techniques were incorporated to highlight the image regions influencing model predictions, thereby improving transparency and facilitating clinical interpretability. A carefully curated dataset was constructed to ensure a comprehensive representation of the morphological variability of endometrial tissues, emphasizing the clinical significance and novelty of the proposed approach.

## Results

To evaluate the effectiveness of the proposed deep learning models in classifying endometrial lesions, we conducted a series of experiments using the curated dataset. The following sections detail the training dynamics, overall performance, and class-specific outcomes.

During the training process, the performance of all four models was monitored using accuracy metrics on the validation set, with EarlyStopping applied to automatically halt training when no further improvement was observed over a predefined number of epochs.

The VGG-based models required a relatively higher number of training cycles to reach stability; for instance, VGG-16 and VGG-19 reached their peak validation performance in the middle stages of training before the EarlyStopping criterion was met. VGG-19, in particular, demonstrated a more consistent alignment between training and validation accuracy, suggesting improved generalization capability.

In contrast, EfficientNet-B0 and DenseNet-121 exhibited a significantly more rapid convergence. DenseNet-121, despite its sophisticated architecture, demonstrated a notably fast learning behavior, reaching its optimal validation performance at a very early stage of the training process. Furthermore, it achieved the lowest loss value among all evaluated architectures, reflecting its superior capability for efficient feature extraction and robust data representation from the outset, even with the specialized nature of the clinical dataset.

The final performance of the four models was evaluated on an independent test set that was not involved in either training or validation. Quantitative results are summarized in Table 1. Overall classification accuracy ranged from 85% to 89%, with DenseNet-121 achieving the highest accuracy (89%), followed by VGGNet-19 (87%), VGGNet-16 (86%), and EfficientNet-B0 (85%).

A class-wise analysis revealed notable differences in model behavior. For endometritis, VGGNet-16 achieved the highest recall (96%), while DenseNet-121 and EfficientNet-B0 demonstrated balanced precision–recall performance (F1-scores of 86% and 80%, respectively). In the normal endometrium class, VGGNet-16 and VGGNet-19 showed strong performance, achieving recall values of 90% and 94%, respectively, with corresponding F1-scores exceeding 90%.

In contrast, performance differences were most pronounced in the polyp class. DenseNet-121 achieved the highest recall (93%) and F1-score (93%) for polyp detection, outperforming the other models. EfficientNet-B0 also demonstrated competitive performance for polyps, achieving an F1-score of 91%, while VGG-based models showed lower recall values (71% for VGGNet-16 and 77% for VGGNet-19).

**Fig. 1:**
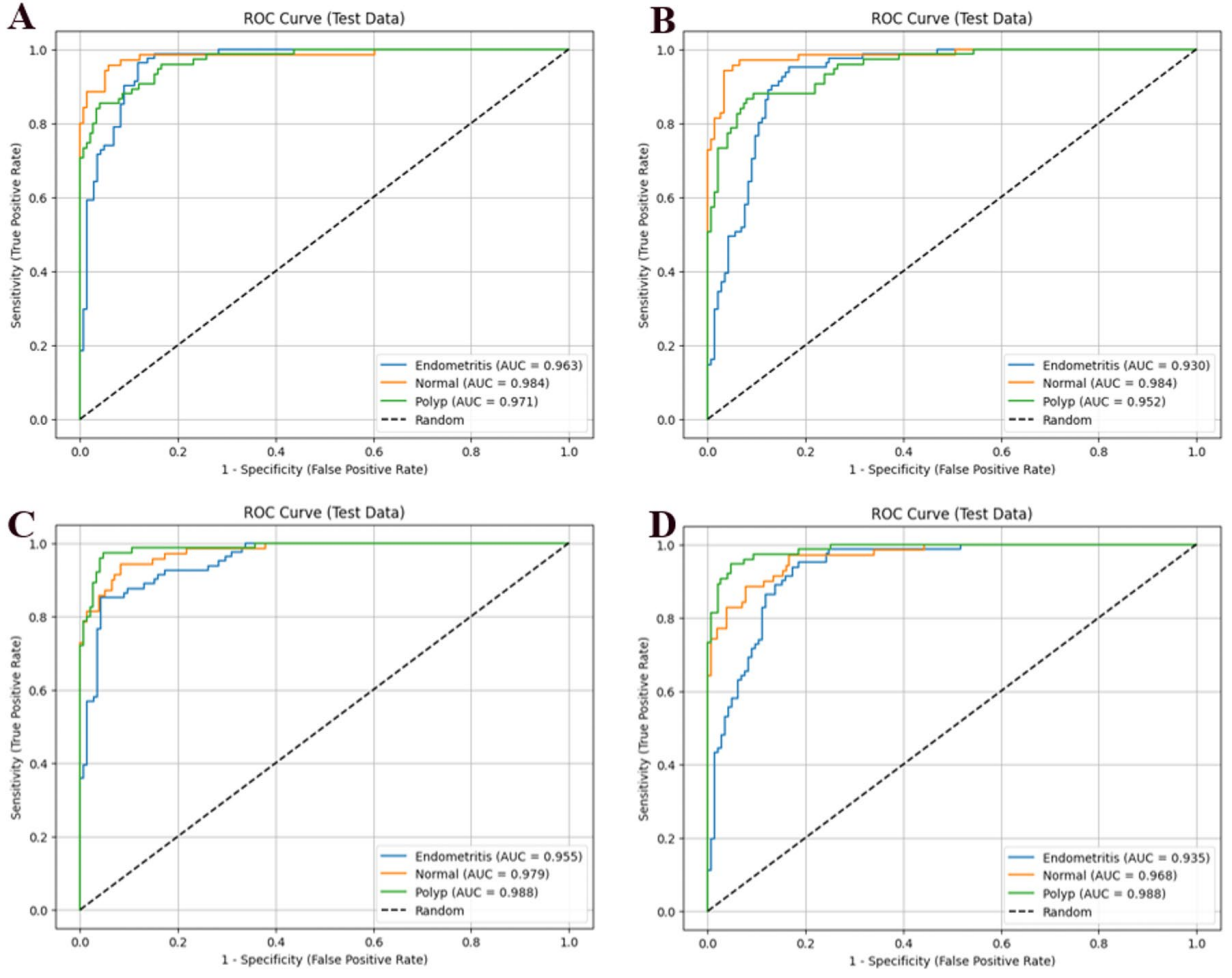
ROC curves of the proposed deep learning models: (**A**) VGG-16, (**B**) VGG-19, (**C**) DenseNet-121, and (**D**) EfficientNet-B0. The Receiver Operating Characteristic (ROC) curves demonstrate the strong diagnostic performance of all four models, with AUC values consistently exceeding 0.93. The proximity of the curves to the top-left corner indicates high sensitivity and specificity for distinguishing between normal, endometritis, and polyp classes. DenseNet-121 and EfficientNet-B0 achieved the highest AUC values for polyp detection (0.988), while VGG-16 showed the highest AUC for normal endometrium (0.984) and endometritis (0.963).

**Fig. 2:**
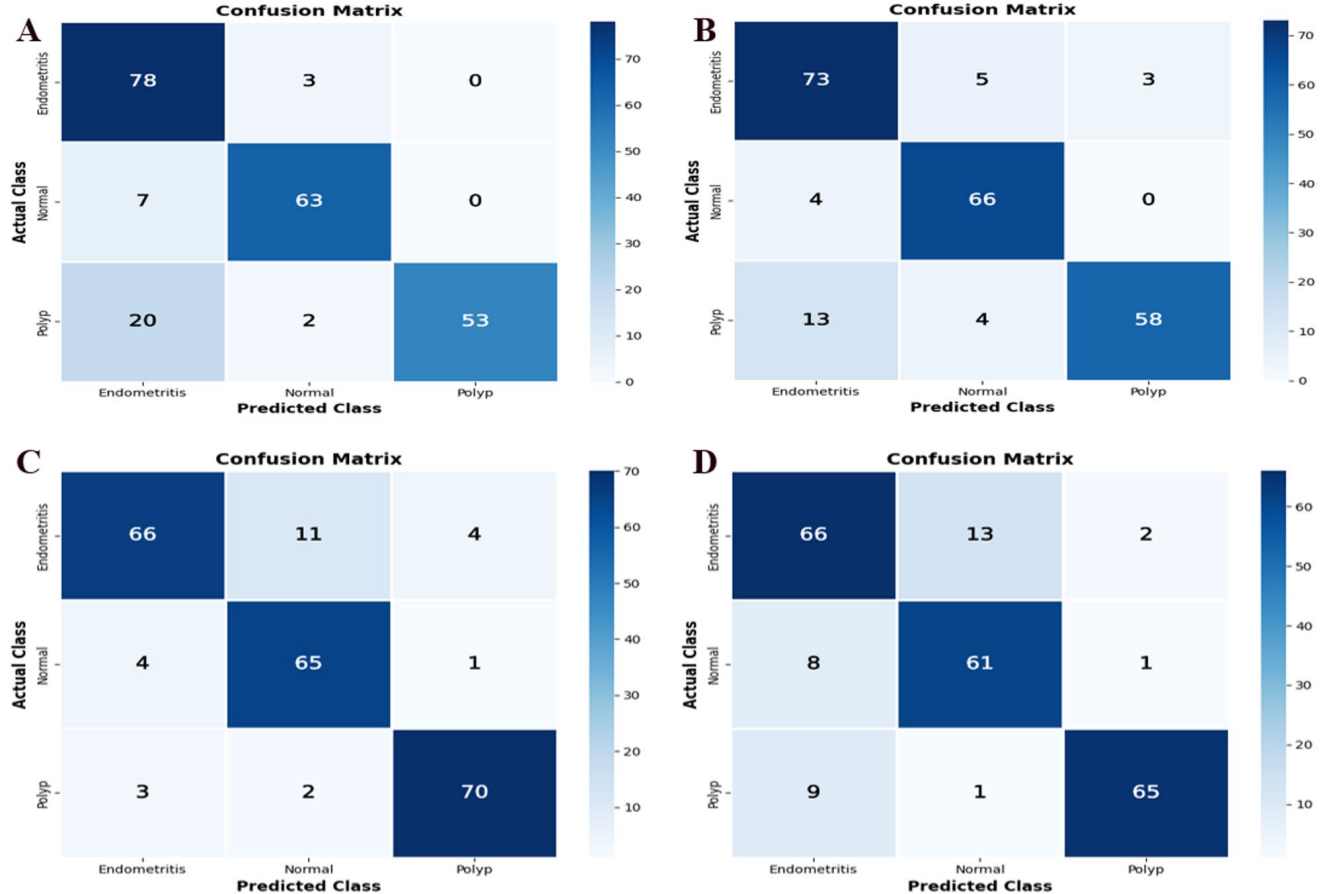
Confusion matrices of the proposed deep learning models: (**A**) VGG-16, (**B**) VGG-19, (**C**) DenseNet-121, and (**D**) EfficientNet-B0. The x-axes represent the predicted labels assigned by the models, whereas the y-axes represent the true labels (Ground Truth). Each cell in the matrix indicates the number of images classified under a specific category; the diagonal cells represent correct predictions where the predicted label matches the true label, while the off-diagonal cells represent misclassifications. This representation allows for detailed assessment of model performance across normal endometrium, endometritis, and polyp classes.

Across all models and classes, high discriminative ability was observed, as reflected by AUC values ranging from 93.0 to 98.8%. DenseNet-121 and EfficientNet-B0 achieved the highest AUC for polyp classification (98.8%), while VGGNet-16 demonstrated strong AUC performance for normal endometrium (98.4%) and endometritis (96.3%). The Receiver Operating Characteristic (ROC) curves for all models are illustrated in Fig. 1, and the corresponding confusion matrices highlighting class-wise prediction patterns are presented in Fig. 2.

**Table 1:** Evaluation results of the proposed deep learning models on the test set.

Model	Precision (%)	Recall (%)	Specificity (%)	f1-score (%)	AUC	Accuracy (%)
VGGNet-16						
Endometritis	74	96	81	84	96.3 (95% CI 0.940–0.982)	86
Normal	93	90	97	91	98.4 (95% CI 0.963–0.997)	
Polyp	100	71	100	83	97.1(95% CI 0.950–0.987)	
VGGNet-19						
Endometritis	81	90	88	85	93 (95% CI 0.895–0.960)	87
Normal	88	94	94	91	98.4 (95% CI 0.965–0.996)	
Polyp	95	77	98	85	95.2 (95% CI 0.924–0.974)	
DenseNet-121						
Endometritis	90	81	95	86	95.5 (95% CI 0.929–0.989)	89
Normal	83	93	92	88	97.9 (95% CI 0.978–0.999)	
Polyp	93	93	97	93	98.8 (95% CI 0.970–0.994)	
EfficientNet-B0						
Endometritis	80	81	88	80	93.5 (95% CI 0.902–0.961)	85
Normal	81	87	91	84	96.8 (95% CI 0.946–0.986)	
Polyp	96	87	98	91	98.8 (95% CI 0.977–0.997)	

### Grad-CAM visualization results

**Fig. 3:**
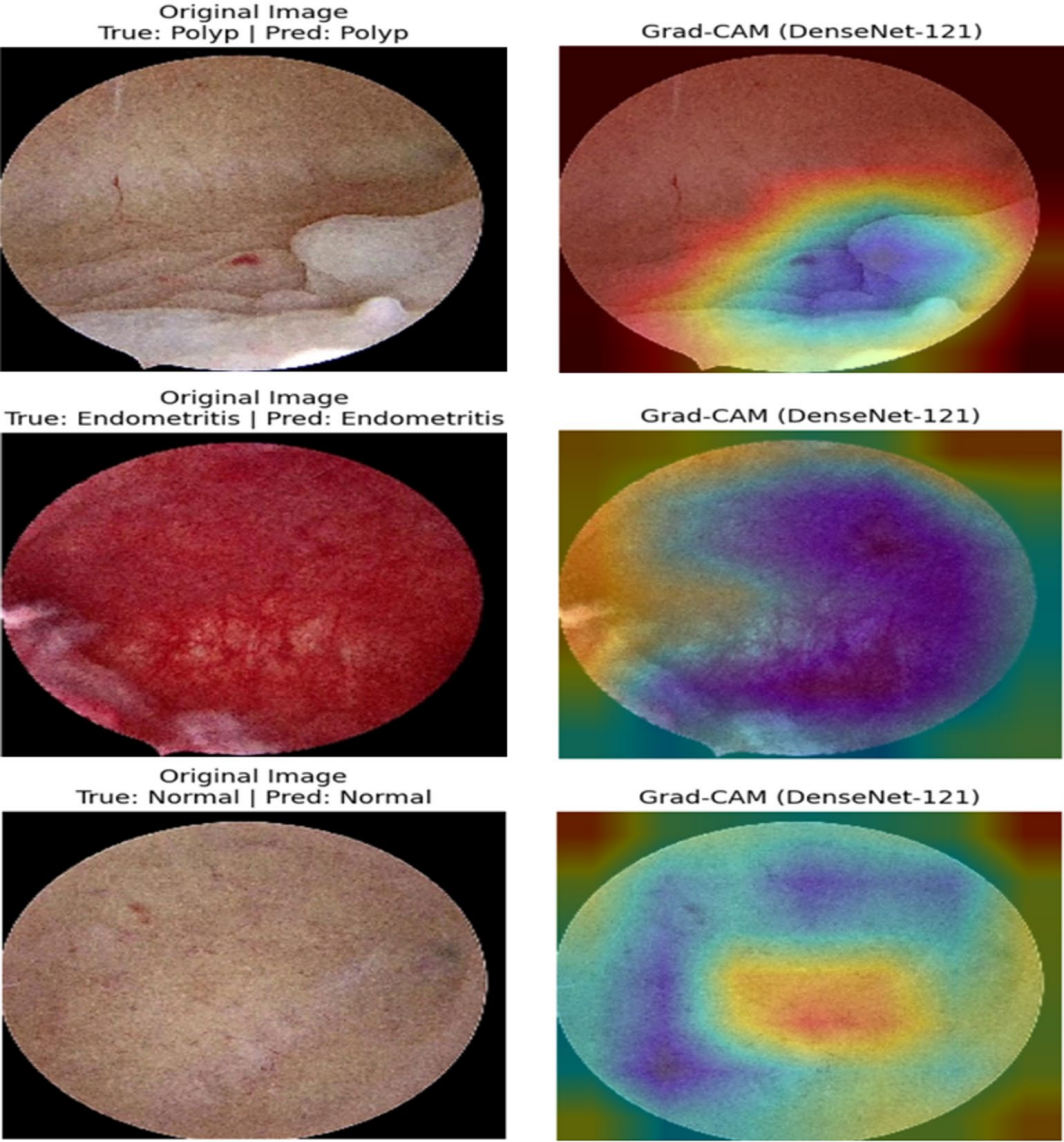
Grad-CAM visualization on hysteroscopic images. Grad-CAM (Gradient-weighted Class Activation Mapping) was applied to highlight the regions within hysteroscopic images that most influenced the model’s final classification. The heatmaps reveal texture and structural patterns used to differentiate between normal endometrium, endometritis, and polyps. The model’s attention aligns closely with clinical knowledge, focusing on tissue protrusions in polyp cases and characteristic surface alterations in endometritis cases. This correspondence confirms that the model’s predictions are based on relevant pathological features.

The qualitative evaluation using Grad-CAM demonstrated that the models successfully focused on characteristic patterns associated with endometrial lesions. As illustrated in Fig. 3, which presents representative examples of heatmaps generated by the DenseNet-121 model, the model’s attention was precisely localized on tissue protrusions in polyp cases and on surface alterations in endometritis cases. This alignment highlights the discriminative regions that contributed most to the correct classifications and provides visual evidence of the model’s diagnostic accuracy.

## Discussion

Recent studies have highlighted the growing potential of deep learning techniques for improving the diagnostic performance of hysteroscopic image analysis. For example, Zhang Y et al. developed a deep learning model based on VGGNet-16 to classify different endometrial lesions using hysteroscopic images with histopathological confirmation, demonstrating that artificial intelligence systems can achieve diagnostic performance comparable to that of experienced clinicians^15^.Similarly, Raimondo D et al. proposed a ResNet-50-based framework for automated classification of endometrial lesions from hysteroscopic images and showed that combining image analysis with clinical variables could further improve diagnostic performance^16^.

Other studies have focused on the automated detection of specific intrauterine lesions. For instance, Zhao A et al. developed a real-time detection system based on the YOLOX algorithm for identifying endometrial polyps from hysteroscopic video frames^9^. In addition, Takahashi Y et al. proposed a deep learning system using multiple convolutional neural networks, including Xception, MobileNetV2, and EfficientNet-B0, to detect endometrial cancer from hysteroscopic images and videos, demonstrating that incorporating temporal video analysis can further enhance diagnostic performance^14^.

The VGGNet-16 and VGGNet-19 models demonstrated a clear superiority in classifying normal endometrium and endometritis, with VGGNet-16 achieving a recall of 96% for endometritis and 90% for normal endometrium. This indicates the ability of these models to effectively capture the general visual characteristics of healthy endometrial tissue as well as the widespread inflammatory features observed in hysteroscopic images (Table 1).

In contrast, the VGG-based models showed a relatively weaker performance in identifying endometrial polyps, with recall values decreasing to 71% for VGGNet-16 and 77% for VGGNet-19. This limitation was clearly reflected in the confusion matrices, which revealed frequent misclassification between the polyp and endometritis classes (Fig. 2).

On the other hand, DenseNet-121 emerged as the best-performing model in the classification of endometrial polyps, which represent the most clinically relevant class in the context of patients undergoing in vitro fertilization (IVF) programs. The model achieved a recall of 93% for the polyp class, along with a high AUC value of 98.8% and a remarkable specificity of 97%, outperforming the other evaluated models in this category.

Although DenseNet-121 achieved the highest performance across several evaluation metrics, formal statistical comparisons between models were not performed; therefore, the observed differences should be interpreted cautiously and may not necessarily indicate statistically significant superiority.

This superior performance highlights the ability of the DenseNet architecture to exploit its dense connectivity mechanism for capturing fine-grained and localized features associated with endometrial polyps, even in cases where visual characteristics overlap with other lesion types. This advantage was observed despite its relatively lower performance in classifying endometritis compared with the VGG-based models.

The observed overlap between endometrial polyps and endometritis aligns with clinical reality, as these conditions frequently coexist or exhibit overlapping hysteroscopic features. Although the gold standard for diagnosing chronic endometritis (CE) is histopathological confirmation via endometrial biopsy^17^, in this study, cases of endometritis were classified based on the clinician’s visual assessment during hysteroscopy regardless of the specific subtype or histopathological classification. This methodological choice was made to reflect the clinical purpose of hysteroscopy as a pre-ART evaluation rather than as a definitive diagnostic procedure. The hysteroscopic features considered suggestive of inflammation such as stromal edema, focal or diffuse hyperemia were used to guide classification. Previous studies have indicated that while these visual features may suggest CE, hysteroscopic examination alone achieves limited diagnostic accuracy (~ 67%) and cannot replace biopsy-based confirmation^18^. Therefore, although our classification reflects real-world clinical practice and the information typically available during pre-ART assessment, the results should be interpreted with caution, particularly regarding the precise histopathological subtype of endometritis.

Despite the inherent limitations of relying solely on visual assessment for endometritis classification, the evaluated deep learning models demonstrated promising performance in detecting inflammatory features. Overall, the models achieved high recall and balanced F1-scores indicating strong sensitivity and reliable classification of endometritis based on hysteroscopic features. These results compare favorably with previously reported studies, which highlighted the limited diagnostic accuracy of hysteroscopic evaluation alone (~ 67%), and suggest that deep learning approaches hold significant potential for enhancing diagnostic reliability, particularly if trained on datasets with biopsy-confirmed labels.

Upon analyzing the misclassified images, a limited number of cases were found to be consistently misclassified across all four models, suggesting that the source of error is not attributable to a specific network architecture. Rather, this behavior reflects intrinsic visual ambiguity and morphological overlap within these cases. Such images are likely to represent borderline cases, sharing visual characteristics across multiple pathological categories. This ambiguity is further amplified by the absence of histopathological confirmation and the reliance on static hysteroscopic images, which deprives the models of the dynamic visual cues that clinicians typically exploit during real-time examination.

Grad-CAM visualizations demonstrated that the model predominantly focused on clinically relevant anatomical regions within the endometrium, such as tissue protrusions characteristic of endometrial polyps and surface alterations associated with endometritis. These regions are consistent with the visual cues commonly relied upon by clinicians during diagnostic decision-making (Fig. 3). Such alignment between model attention and clinical reasoning enhances the interpretability of the predictions and supports the potential use of these models as explainable decision-support tools in clinical practice, though external validation on larger multi-center datasets remains necessary before clinical deployment.

The achieved performance was obtained using deep learning models with minimal architectural modifications, relying on transfer learning techniques rather than full training from scratch. The high quality of the hysteroscopic images—extracted from 4 K-resolution videos—likely played a significant role in enhancing the models’ ability to capture fine-grained features, particularly for endometrial polyps, which require the identification of subtle and localized tissue details that may be lost in lower-resolution images^19^.

This study gains its significance from being—to the best of our knowledge—the first to systematically evaluate the DenseNet-121 architecture for hysteroscopic image classification, specifically focusing on lesions that directly impact implantation success in IVF patients. Our findings reveal that DenseNet-121 offers a superior ability to capture subtle morphological details through its dense connectivity mechanism. Notably, this study is also among the first to explore the automated detection of endometritis, achieving high diagnostic performance in identifying inflammatory changes that are often subtle and difficult to categorize. The high clarity of the source hysteroscopic videos, combined with DenseNet’s feature-reuse capability, allowed for capturing fine-grained patterns in both polyps and endometritis—features that may have been overlooked in earlier studies using different architectures. These findings highlight the promise of our approach as a decision-support tool in assisted reproductive technology, though broader clinical applicability will require validation across multiple centers and with larger, more diverse datasets.

## Limitation

Despite the promising performance of the proposed models, several limitations should be acknowledged. First, the dataset was collected from a single fertility center, which may limit the generalizability of the findings to other clinical settings or imaging systems. Second, although the dataset included representative hysteroscopic images, the total number of cases remained relatively limited, which may restrict the broader generalization of the results.

Another limitation is the absence of demographic and clinical metadata, such as age, BMI, hormonal status, cycle phase, and prior IVF attempts, which were not collected as part of the study protocol. Consequently, assessment of potential confounding factors, patient heterogeneity, and subgroup-specific model performance was not possible. Future studies incorporating multimodal clinical data may provide a more comprehensive evaluation of model robustness and generalizability.

A further limitation concerns the reference standard used for endometritis classification. Cases were labeled based on hysteroscopic visual features suggestive of inflammation, without histopathological confirmation. As reported by Song et al., hysteroscopic evaluation alone achieves a diagnostic accuracy of approximately 67% for chronic endometritis^18^. Therefore, the models trained in this study learn to replicate expert visual judgment rather than a pathological gold standard. Future studies should incorporate biopsy-confirmed labels to establish a more rigorous reference standard and further validate model performance.

Finally, while the models demonstrated strong performance in image classification, the study relied on static images extracted from videos rather than full temporal video analysis. Future studies incorporating larger multi-center datasets and real-time video-based deep learning models may further enhance the robustness and clinical applicability of automated hysteroscopic diagnosis.

## Conclusion

In this study, we applied and evaluated four deep learning models for the classification of endometrial lesions using images extracted from high-resolution (4 K) hysteroscopic videos in the context of assisted reproductive technology (IVF) patients. The models demonstrated promising performance in distinguishing between normal endometrium, endometrial polyps, and endometritis, highlighting their potential as supportive tools for visual assessment during hysteroscopy while complementing—rather than replacing—clinical decision-making. While these results are encouraging, it is important to acknowledge that this work represents a preliminary investigation conducted at a single center, with endometritis classified through visual hysteroscopic assessment rather than histopathological confirmation.

Future work would focus on integrating hysteroscopic images with clinical and histopathological data, as well as transitioning from static image analysis to full video-based processing to leverage dynamic visual information. Additionally, expanding the dataset and refining training strategies are expected to further improve model generalizability and facilitate the integration of such systems into real-world clinical settings.

Overall, this study highlights the promise of deep learning as an explainable decision-support tool in reproductive medicine, with potential for integration into clinical workflows after further multi-center validation.

## Methods and materials

### Dataset

The training and testing data were prospectively collected from patients enrolled in assisted reproductive technologies (ART) programs (i.e. in vitro fertilization) who underwent hysteroscopy at Orient Fertility and Genetics Hospital in Damascus. Data acquisition spanned six months, from August 2024 to January 2025. Hysteroscopic procedures were recorded concurrently during the interventions, following informed consent obtained from all participants.

All videos were captured using a high-definition 4 K hysteroscopic system, equipped with a state-of-the-art 4 K hysteroscopic camera from KARL STORZ (Germany). The system produced images at a resolution of 2160 × 3840 pixels, exceeding 8 million pixels per frame.

All collected data were handled in accordance with patient confidentiality and privacy regulations, and all personally identifiable information was removed. All experiments conducted in this study were performed in accordance with the guidelines of the Ethical Committee for Biomedical Research at Damascus University, Syria. The experimental protocols received approval under license number FMEE-051124-346 from the same committee.

**Fig. 4:**
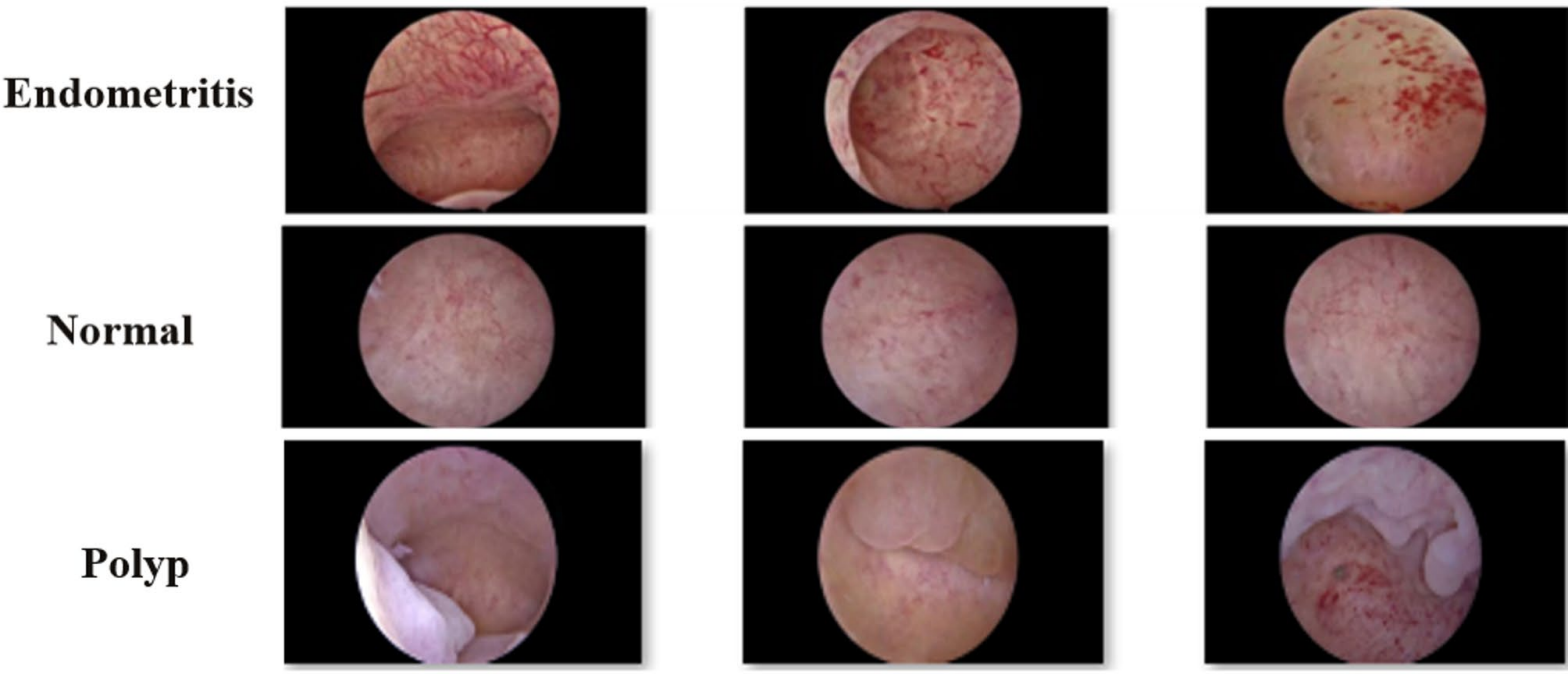
Examples of the hysteroscopic images used in this study.

Following data collection, a total of 250 videos were obtained. All videos underwent an initial review to annotate the observed pathological conditions. The review revealed that the most common findings among patients undergoing IVF at the hospital were endometrial polyps and endometritis. Consequently, the scope of this study was defined to focus on these two conditions due to their high prevalence and clinical relevance. Representative examples of endometrial images from the dataset used in this study are presented in Fig. 4.

Endometritis cases were included in the dataset as they represent a relatively frequent intrauterine condition among patients undergoing IVF programs. The dataset did not distinguish between specific subtypes of endometritis due to the limited number of available cases. Therefore, the analysis considered endometritis as a general inflammatory condition of the endometrium. Diagnostic labels were assigned by experienced clinicians based on hysteroscopic visual assessment and established hysteroscopic features reported in the literature, such as stromal oedema, focal or diffuse endometrial hyperaemia, the “strawberry” appearance of the endometrium, and the presence of micropolyps. This approach ensured that the extracted images reflected clinically relevant morphological characteristics of inflamed endometrial tissue. It should be noted that endometritis cases were classified based on hysteroscopic features suggestive of inflammation; no histopathological confirmation was obtained.

Conversely, videos containing congenital uterine anomalies, uterine fibroids, or low-quality and blurred images were excluded to ensure dataset homogeneity and improve analytical accuracy. Based on these criteria, a total of 200 hysteroscopic video recordings were ultimately included in the study, encompassing cases of endometritis, endometrial polyps, and normal endometrium.

The video recordings were segmented into static images, resulting in a total of 1,500 images. Image selection and labeling were performed manually by expert clinicians. The dataset was then divided into three independent subsets (training, validation, and testing) with proportions of 70%, 15%, and 15%, respectively (Table 2 shows the details of the dataset split).

Multiple images were selected from each patient to accurately represent the visual morphological variations of the endometrium and the observed lesions. The dataset was split at the patient level: all images from a given patient were assigned exclusively to the training, validation, or test set, ensuring no patient appeared in more than one subset. The detailed distribution of patients and images across all splits and categories is presented in Table 2.

**Table 2:** Partition details of the endometrial lesion dataset for classification.

Category	Dataset		Training set		Validation set		Test set	
	No. of patients	No. of images	No. of patients	No. of images	No. of patients	No. of images	No. of patients	No. of images
Normal	60	466	42	326	9	70	9	70
Polyp	57	495	40	345	8	75	9	75
Endometritis	83	539	58	377	12	81	13	81
Total	200	1500	140	1048	29	226	31	226

### Data preprocessing

To have the models focus on the clinically relevant regions and enhance computational efficiency, an initial manual cropping phase was performed under expert medical supervision. This step ensured the removal of non-informative black borders and artifacts while centering the circular hysteroscopic field of view on the endometrial tissue.

Following cropping, online data augmentation was dynamically applied during the training phase using ImageDataGenerator. This strategy enabled the generation of image variants at each training epoch without increasing the physical size of the stored dataset. To preserve the essential anatomical characteristics of endometrial lesions, the augmentation pipeline was configured with controlled transformation parameters, including limited rotations (± 10 degrees), horizontal and vertical spatial shifts restricted to 5% of the image dimensions, zoom operations within a 5% range, random horizontal flipping, and brightness adjustments within the range [0.8, 1.2] to simulate variations in illumination conditions while maintaining diagnostic integrity.

For normalization, all pixel intensity values were rescaled by a factor of 1/255. Importantly, data augmentation was strictly excluded from the validation and test sets; these sets underwent only cropping, rescaling, and resizing to ensure an unbiased evaluation of model performance on unaltered data.

**Fig. 5:**
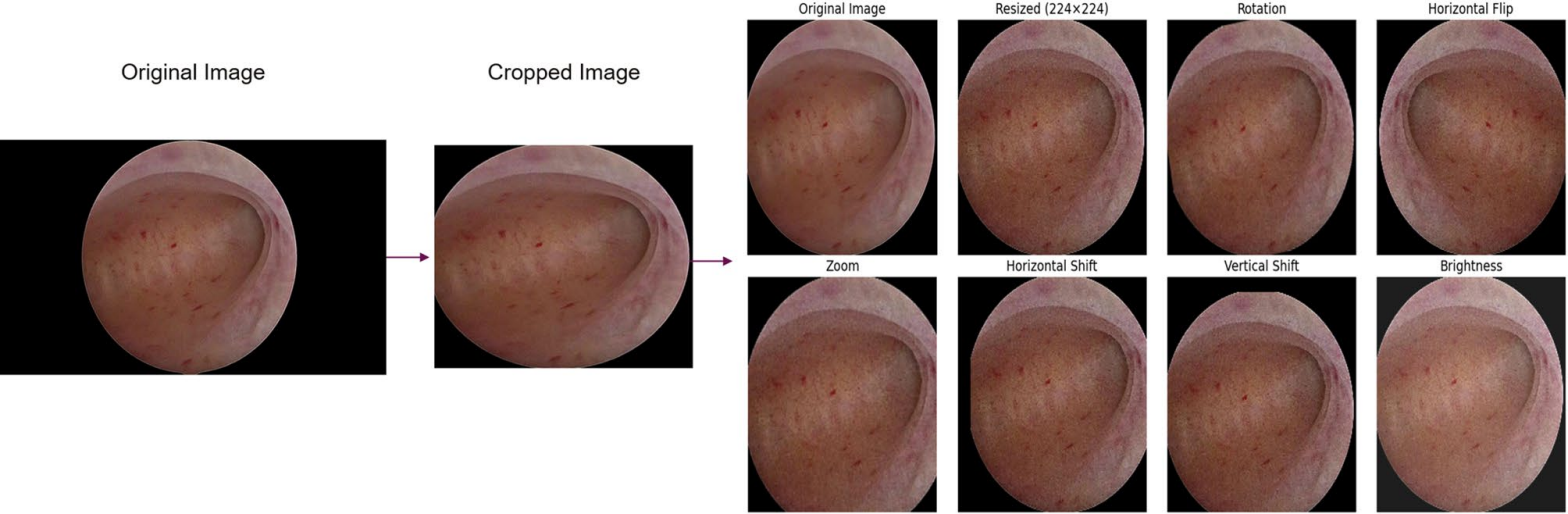
Example of image preprocessing.

Finally, all images were resized to 224 × 224 pixels to comply with the architectural requirements of the selected deep learning models. An example of the applied data augmentation transformations is shown in Fig. 5.

### Convolutional neural networks and transfer learning

Transfer learning allows a pre-trained model to be adapted for a related task, leveraging previously learned representations and architectures from large datasets like ImageNet. By replacing the original fully connected layers with task-specific layers while retaining the preceding feature-extracting layers, this approach accelerates training, improves performance, and reduces the need for large annotated datasets^20^.

**Fig. 6:**
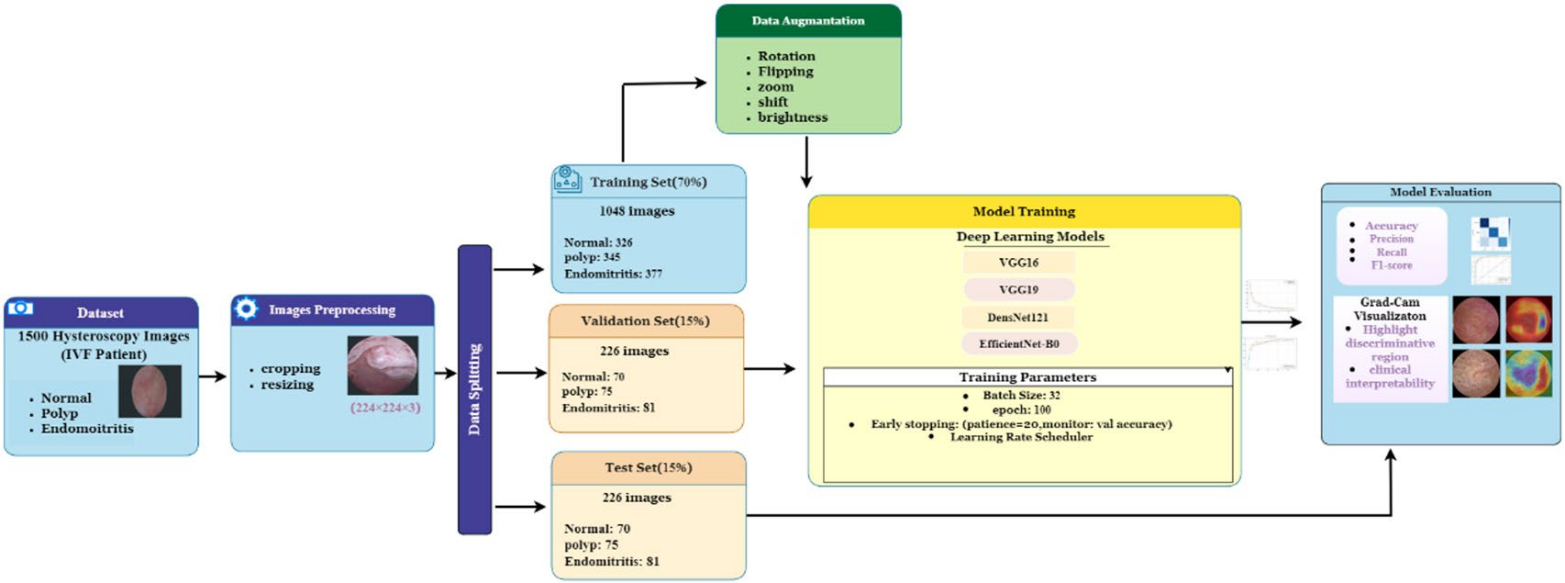
Proposed workflow of the study.

In this study, VGG-16, VGG-19, DenseNet-121, and EfficientNet-B0 were employed to classify high-resolution hysteroscopic images of the endometrium from IVF patients. All models were initially pre-trained on ImageNet and subsequently fine-tuned on the study dataset to adapt to the endometrial lesion classification task. The overall framework is illustrated in Fig. 6.

VGG-16 and VGG-19 are deep convolutional networks developed by the Oxford Visual Geometry Group. They use small convolutional filters and deep architectures to extract fine-grained features. VGG-16 has fewer layers than VGG-19, resulting in faster inference and lower memory requirements, while performance differences are minimal^21,22^.

DenseNet-121 connects each layer to all preceding layers, enhancing feature propagation and alleviating the vanishing gradient problem. Its four dense blocks (6, 12, 24, and 16 layers) make it highly effective for medical image classification, particularly with limited training data^23^.

EfficientNet-B0 employs MBConv blocks with Squeeze-and-Excitation modules, depthwise separable convolutions, and inverted residuals to balance accuracy and computational efficiency. Its compound scaling strategy optimizes network depth, width, and input resolution, making it suitable for high-resolution medical imaging^24^.

### Model generation and training using transfer learning

**Fig. 7:**
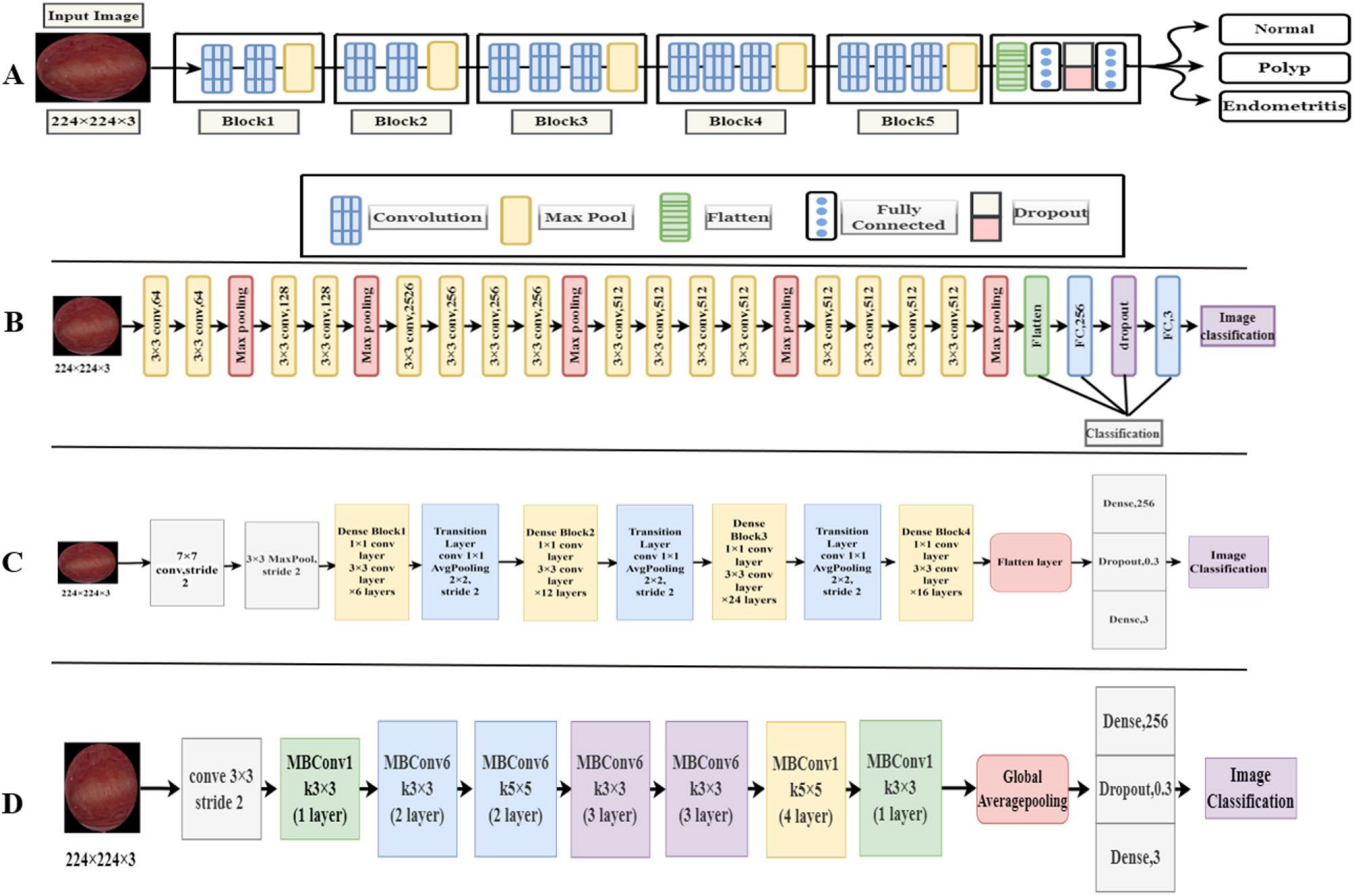
Architecture of the modified deep learning models used in this study. Each panel shows the full architecture of a pretrained convolutional neural network modified for hysteroscopic endometrial image classification: (**A**) VGG-16, (**B**) VGG-19, (**C**) DenseNet-121, and (**D**) EfficientNet-B0. The original convolutional backbones are retained, and the fully connected layers removed from the pretrained models were replaced with a dense layer (256 neurons), a dropout layer (0.3), and a final Softmax layer to perform three-class classification (polyps, endometritis, and normal endometrium).

In this study, transfer learning was applied to four pretrained convolutional neural network architectures, namely VGG-16, VGG-19, DenseNet-121, and EfficientNet-B0. For each model, the original fully connected layers were removed, and the convolutional base was retained as a feature extractor to construct the new classification architectures^25^. To transform the extracted features into classification vectors, a Flatten layer was implemented for the VGG-16, VGG-19, and DenseNet-121 models. Conversely, Global Average Pooling (GAP) was integrated into the EfficientNet-B0 architecture to align with its advanced scaling design and to substantially reduce the number of trainable parameters^26^. The modified structural configurations for all four models are further illustrated in Fig. 7. A fully connected dense layer consisting of 256 neurons was added on top of each backbone network, followed by a dropout layer with a rate of 0.3 to mitigate overfitting^27^.

The Softmax activation function was employed in the final layer of each model to classify hysteroscopic endometrial images into three categories: polyps, endometritis, and normal endometrium. All models were trained using the same image dataset and for an identical number of epochs. An EarlyStopping strategy was applied to monitor the model performance on the validation set, where training was automatically halted if no improvement was observed for 20 consecutive epochs (patience = 20), with restoration of the best-performing model weights.

Furthermore, the ReduceLROnPlateau scheduler was employed to dynamically adjust the learning rate with a reduction factor of 0.3. Based on empirical experiments, an initial learning rate of $\:{1\times\:e}^{-5}$ was established for the VGG and DenseNet models to ensure training stability. In contrast, EfficientNet-B0 required a higher initial learning rate of $\:{1\times\:e}^{-4}$ to achieve optimal convergence and performance. All models were optimized using the Adam optimizer, with the Cross-Entropy loss function utilized for the multi-class image classification task.

### Evaluation metrics

To evaluate the performance of the proposed models, a set of commonly used evaluation metrics in medical image classification was employed, including accuracy, precision, recall, and F1-score. These metrics provide a comprehensive assessment of the models’ ability to discriminate between the different classes.

In addition, confusion matrices were utilized to perform a detailed analysis of model performance and to identify patterns of misclassification among the classes. All evaluation metrics were computed exclusively on the test set, which was not used during either training or validation, in order to ensure an objective and unbiased assessment of the models’ generalization performance.

The evaluation metrics were calculated according to the following equations:

Accuracy: Measures the proportion of correctly predicted instances among the total number of cases.

1 $$\mathrm{Accuracy}=\:\frac{(\mathrm{T}\mathrm{P}+\mathrm{T}\mathrm{N})}{\:(\mathrm{T}\mathrm{P}+\mathrm{T}\mathrm{N}+\mathrm{F}\mathrm{N}+\mathrm{F}\mathrm{P})}$$

Recall (Sensitivity): Reflects the model’s ability to correctly identify all relevant cases.

2 $$\mathrm{Recall}=\:\frac{\mathrm{T}\mathrm{P}}{\:(\mathrm{T}\mathrm{P}+\mathrm{F}\mathrm{N})}$$

Precision: Indicates the accuracy of positive predictions.

3 $$\mathrm{Precision}=\:\frac{\mathrm{T}\mathrm{P}}{\:(\mathrm{T}\mathrm{P}+\mathrm{F}\mathrm{P})}$$

F1-score: Represents the harmonic mean of Precision and Recall, providing a balanced measure particularly in cases of class distribution variance.

4 $${\text{F1 - score}} = \:\frac{{2 \times \:\:{\mathrm{Precision}}\: \times \:\:{\mathrm{Recall}}}}{{({\mathrm{Recall}}\: + \:{\mathrm{Precision}})}}$$

Specificity: Measures the proportion of actual negatives correctly identified by the model.

5 $$\mathrm{Specificity}=\:\frac{\mathrm{T}\mathrm{N}}{\:(\mathrm{T}\mathrm{N}+\mathrm{F}\mathrm{P})}$$

where TP, TN, FP, and FN represent true positives, true negatives, false positives, and false negatives, respectively.

In addition to the conventional evaluation metrics, Receiver Operating Characteristic (ROC) curves and the Area Under the Curve (AUC) were employed to assess the discriminative ability of the models across the different classes.

AUC values were calculated based on the test set and were used as an additional indicator of classification performance, as higher AUC values reflect a stronger ability of the model to distinguish between correctly and incorrectly classified images.

### Model interpretability (Grad-CAM)

To elucidate the decision-making process within the proposed models and to provide a visual interpretation of the classification outputs, explainable artificial intelligence (XAI) techniques were employed, representing a crucial step toward enhancing transparency and trust in medical deep learning systems^28^. These techniques enable the identification of image regions within the endometrium that have a direct influence on the model’s predictions, thereby allowing clinicians to verify that the model relies on clinically and anatomically relevant features when distinguishing between different lesions.

In this study, Grad-CAM^29^ was applied to generate visual heatmaps highlighting the discriminative regions that contributed most to the model’s predictions in correctly classified images.

### Computational environment and frameworks

All deep learning models were implemented using the Python programming language and executed on the Google Colaboratory (Colab) cloud platform. To leverage high-performance computing capabilities, GPU acceleration (NVIDIA Tesla T4) was employed, ensuring efficient training and fine-tuning of the proposed architectures. Model development, training, and evaluation were conducted using the TensorFlow and Keras deep learning frameworks. For data handling, statistical analysis, and visualization, standard scientific libraries including NumPy, Pandas, and Matplotlib were utilized. This standardized computational environment was maintained across all experiments to ensure consistency, reliability, and reproducibility of the results.

## Data Availability

The datasets generated during and/or analyzed during the current study are available from the corresponding author on reasonable request.
